# Cardiac risk factors and events in patients with prostate cancer commencing androgen deprivation therapy: analysis from a tertiary care centre in the Middle East

**DOI:** 10.3332/ecancer.2022.1445

**Published:** 2022-09-14

**Authors:** Mona Ali Hassan, Talar Telvizian, Mostafa Abohelwa, Deborah Mukherji, Hadi Skouri

**Affiliations:** 1 Division of Hematology/Oncology, American University of Beirut Medical Center, Beirut 1107 2020, Lebanon; 2 Department of Internal Medicine, Lankenau Medical Center, Wynnewood, PA 19096, USA; 3 Department of Internal Medicine, Texas Tech University Health Science Center, Lubbock, TX 79430, USA; 4 Division of Cardiology, American University of Beirut Medical Center, Beirut 1107 2020, Lebanon

**Keywords:** androgen deprivation therapy, ADT, cardiovascular risk factors, cardiovascular events, Middle East, prostate cancer

## Abstract

**Background:**

Androgen deprivation therapy (ADT) is the mainstay of treatment for advanced prostate cancer, improving symptoms and prolonging survival. There is an association between ADT use and cardiovascular (CV) events, particularly in patients with preexisting risk factors. In men diagnosed with prostate cancer, CV disease is the principal non-cancer-related cause of death. There are no definite guidelines to stratify patients based on CV risk prior to ADT initiation. This is the first study on cardiac risks and events in patients with prostate cancer treated with ADT from the Middle East region, a population known to have a high prevalence of CV risk factors.

**Results:**

A retrospective study of 234 patients with prostate cancer, who received ADT therapy at a tertiary care centre in Lebanon was conducted. CV risk factors at baseline and CV events on ADT were reviewed. The median age was 68 years (48–92 years). The majority of patients had stage 4 diseases at diagnosis (49.6%) with a median duration of 12 months on ADT. In our cohort, 24.4% had body mass index > 30, 52.1% had smoking history, 25.6% were diabetic, 19.7% had history of coronary artery disease, 9.8% had heart failure history and 52.9% had hypertension. Less than half of the patients had a documented lipid profile at baseline. Twenty-two patients (9.5%) had documented cardiac events following ADT initiation.

**Conclusions:**

In this cohort of patients from the Middle East, we found that one third of the population had established coronary artery disease at baseline and 9.5% had documented cardiac events on ADT initiation. Our study highlights the gaps in CV risk assessment for this high-risk group of patients with prostate cancer in addition to high prevalence of CV comorbidities. Risk and resource-stratified algorithms are needed before starting ADT therapy for optimal CV health. Increased awareness, collaboration and referral mechanisms between oncologists, urologists and cardiologists are also needed to provide optimal care.

## Background

Prostate cancer is the most common cancer affecting men worldwide with an average age of 67 years at diagnosis [1]. Androgen Deprivation Therapy (ADT) is the cornerstone of treatment for advanced stages of prostate cancer and is also used for localised disease in combination with radiation therapy [2].

Despite the fact that ADT prolongs survival in advanced prostate cancer, several studies have indicated that men receiving ADT are at increased risk of fatal and nonfatal cardiovascular (CV) events [3]. This risk is particularly high in men with CV risk factors or previous CV events [4, 5]. So far, there are no specific guidelines for baseline CV risk stratification of patients diagnosed with prostate cancer prior to initiation of ADT. Men in the Middle East have a high prevalence of CV disease [6] and data suggest that a high proportion of men diagnosed with prostate cancer in the region present with advanced disease at diagnosis, necessitating the long-term use of ADT [7]. Our study is the first to evaluate cardiac risks and events in patients on ADT from Lebanon and the Middle East, a population known to have a high prevalence of CV risk factors and our main objective is to look at the baseline characteristics of our cohort and the incidence of CVS among them.

## Methods

Following Institutional Review Board approval, data were collected through a retrospective chart review of all patients with prostate cancer, who were initiated on ADT from 2014 to 2019 at the American University of Beirut Medical Center. The parameters collected include: prior symptomatic coronary artery disease including carotid artery disease or peripheral artery disease (e.g. stable angina, acute myocardial infarction, transient ischaemic attacks, stroke, ischaemic claudication), age, gender, height, weight, smoking status (current, ex- or non-smoker), history of chronic kidney disease, history of systemic inflammatory disease (e.g. rheumatoid arthritis, psoriasis), family history of premature CV disease (before age 60 years). In addition, the following parameters pre and post ADT initiation were collected: lipid profile, diabetes mellitus, cardiac arrhythmia, heart failure and the development of cardiac events and death on ADT therapy.

Statistical analysis was performed using SPSS version 25. Descriptive analysis was performed for age, cancer stage, CV risk factors and ADT duration. The prevalence of CV risk factors at baseline and CV events while on ADT therapy was analysed through basic percentage calculation.

## Results

In our cohort, 234 patients received ADT for prostate cancer as a part of their treatment plan over the period studied. The median age at diagnosis was 68 with a range of 48–92 years. The majority of patients had stage 4 disease at diagnosis (49.6%) with a median duration of 12 months on ADT**.** Regarding initial ADT, 17 patients had surgical castration with orchiectomy while the remainder had luteinising hormone-releasing hormone agonist or antagonist. Two patients did not have any documentation about the type of hormonal treatment. Baseline demographic factors and prostate cancer characteristics are included in Table 1, where 142 patients (60.7%) had all the factors documented in the chart. Table 2 demonstrates the baseline CV risk factors, and only 84 patients (35.9%) had a full set of data.

Only 95 patients (40.6%) before ADT and 98 patients (41.8%) after ADT had a lipid profile documented in their chart. The mean values before and after ADT, respectively, were: total cholesterol (174.4 and 179.4 mg/dL), LDL (107.6 and 113.4 mg/dL) and HDL (44.2 and 45.4 mg/dL).

Table 3 highlights the incidence of cardiac risk factors after ADT initiation, the most common diagnosis being dyslipidaemia (11.5%) followed by arrhythmias (10.7%). After ADT initiation, 22 patients (9.4%) had a documented CV event including acute myocardial infarction, acute coronary syndrome or stroke. Eight patients (3.4%) underwent percutaneous coronary intervention or coronary artery bypass grafting during hormonal treatment. Overall, 32 patients (9.8%) were documented to have died during treatment.

## Discussion

The association between ADT and CV events was first demonstrated in 2006 when Keating *et al* [8] conducted a study of over 73,000 Medicare patients with localised prostate cancer which showed increase in the incidence of sudden cardiac death, coronary heart disease, myocardial infarction and diabetes among 36% of men on GnRH agonists. The mechanism by which ADT is thought to increase CV risk is by decreasing insulin sensitivity and promoting dyslipidaemia and obesity, thus leading to an increased risk of CV morbidity and mortality [9].

The Middle Eastern population in general, and Lebanon in particular, is a population that is known for high incidence of CV events occurring in around 13.7% of the overall population [10]. A study conducted in 2010 showed that ischaemic heart disease was the leading cause of death in Lebanon (32.2%) [11].

In our study, we found that one third of the population had established coronary artery disease at baseline and 9.5% had documented cardiac events on ADT initiation and, in this cohort of patients, the median age was 68 years (48–92 years). The majority of patients had stage 4 disease at diagnosis (49.6%) with a median duration of 12 months on ADT. In our cohort, 24.4% had body mass index > 30, 52.1% had smoking history, 25.6% were diabetic, 19.7% had history of coronary artery disease, 9.8% had heart failure history and 52.9% had hypertension. Less than half of the patients had a documented lipid profile at baseline. Twenty two patients (9.5%) had documented cardiac events following ADT initiation.

Recently, the Cardio‐Oncology Study Group of the Heart Failure Association of the European Society of Cardiology in collaboration with the International Cardio‐Oncology Society has published the baseline CV risk stratification in oncology patients receiving cancer therapies. They highlighted the role of risk stratification in prostate cancer patients receiving or need to be initiated on ADTs [12]. Their risk stratification pro forma will divide the patients into high, medium and low risk depending on the presence of preexisting CV disease or on the CV disease 10-year risk score. It was suggested that any of the following risk scores can be used: ESC Heart Score, QRISK, ACC/AHA pooled cohort CV risk calculator or JBS3 risk score. There are essential risk factors and variables that are required on screening prior to initiation of ADT and to help calculate CV risk score. These variables need to be obtained in either the urology or oncology clinic so the risk score can be estimated. Variables needed include: age, gender, ethnic group, height, weight, social class indicator, smoking status (current, ex- or non-smoker), total cholesterol, high-density lipoprotein cholesterol, systolic blood pressure (mmHg), diabetes status, family history of premature CV disease (before 60 years), chronic kidney disease, atrial fibrillation, systemic inflammatory disease (e.g. rheumatoid arthritis, psoriasis). The importance of preventive cardio oncology is rapidly evolving recently especially with multi-modality risk prediction strategies, moving towards precision surveillance and interventions that allow for safe continuation of life-saving chemotherapy [13].

Initially we intended to calculate the atherosclerotic cardiovascular disease risk (ASCVD) score of all patients to stratify them into low, borderline, medium and high risk, but most of the patients did not have any documented lipid profile at baseline before ADT initiation and this was particularly noticed among patients who were followed up by urologist rather than those who were followed up by an oncologist. This is why it is essential to have a unified algorithm among all specialities taking care of these patients to prevent CV complication during long-term therapy.

**Figure 1.**
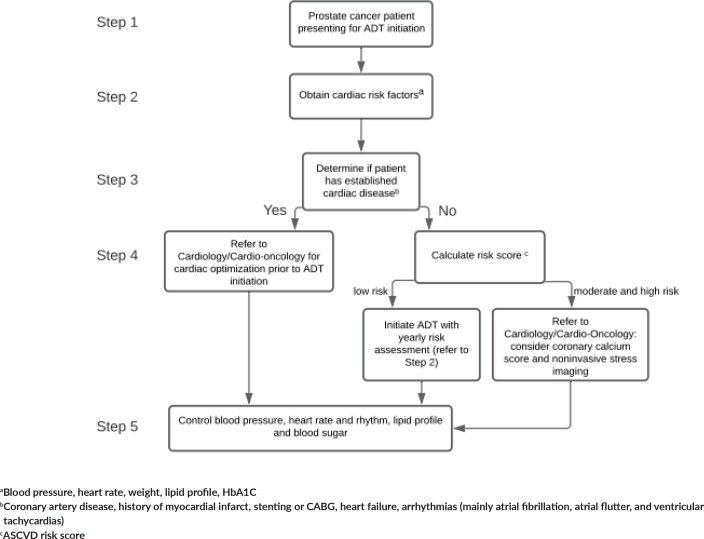
Proposed algorithm for CV risk stratification of prostate cancer patients before ADT initiation.

We are suggesting an algorithm (see Figure 1) for CV risk stratification of prostate cancer patients before ADT initiation, as outlined in Figure 1. ADT for prostate cancer patients is usually a long-term therapy and patients would benefit from counselling about CV effects, baseline risk assessment and timely referral to a cardiologist for the management of their cardiac risk factors and/or diseases.

The limitation of our study lies mainly in the small sample size and missing data in the charts. It is important to note that patients were followed by one or more different speciality physicians including oncologists, urologists, radiation oncologists and cardiologists which likely contributed to missing and decentralised data Also, one of the limitations is that we were not able to do regression analyses (Cox) to look at potential confounders as well as stratified analyses based on the risk factors identified at baseline.

## Conclusions

This is the first study on cardiac risks and events in patients on ADT from Lebanon and the Middle East region, a population which is known to have a high prevalence of CV risk factors. In this cohort of patients from the Middle East, we found that one third of the population had established coronary artery disease at baseline and 9.5% had documented cardiac events with a significant percentage with CV risk factors on ADT initiation. Our study highlights the gaps in CV risk assessment for this high-risk group of patients with prostate cancer. Risk and resource-stratified algorithms are needed before starting ADT therapy for optimal CV health. Increased awareness, collaboration and referral mechanisms between oncologists, urologist and cardiologists are also needed to optimise patient care.

## List of abbreviations

ADT, Androgen deprivation therapy; CV, Cardiovascular.

## Declarations

None.

## Ethical approval and consent to participate

Institutional Review Board approval was obtained.

## Consent for publication

NA.

## Availability of data and material

Data is available upon request.

## Conflicts of interest

DM: Research funding from Astellas, honoraria/travel support from Astellas, Janssen, Bayer, Sanofi.

All other authors: none.

## Funding

None.

## Authors’ contributions

DM and HS: conception and design, supervision

TT: data collection, analysis and results, manuscript writing

MH: data collection, manuscript writing

MA: data collection, manuscript writing.

## Figures and Tables

**Table 1.** Patient nationalities and disease stage at time of diagnosis.

		Frequency *N* = 234	Percent (%)
Nationality	Lebanese	192	82.1
	Iraqi	33	14.1
	Others^a^	9	3.8
Stage at diagnosis	I	2	0.8
	II	32	13.7
	III	35	15
	IV	116	49.6
	Missing	49	20.9
Gleason grade at diagnosis	1	9	3.8
	2	21	9
	3	54	23.1
	4	63	26.9
	5	49	21
	Missing	38	16.2

a Syrian, Jordanian, Palestinian

**Table 2.** Baseline CV risk factors.

	Frequency (*N*)	Percent (%)
Obese (BMI > 30)	57	24.4
Ever smoker	122	52.1
Alcohol (>3 units/day)	10	4.3
Hypertension	124	52.9
Diabetes	60	25.6
Dyslipidaemia	89	38.0
Chronic kidney disease	36	15.4
Coronary artery disease	46	19.7
Heart failure	23	9.8
Atrial fibrillation	18	7.6
Family history of CV disease before age 50 years	2	0.85
Systemic inflammatory disease (e.g. rheumatoid arthritis, psoriasis)	5	2.1

**Table 3.** Incidence of cardiac risk factors after ADT initiation.

	Frequency (N)	Percent (%)
Coronary artery disease	13	5.6
Diabetes mellitus	15	6.4
Dyslipidaemia	27	11.5
Heart failure	15	6.4
Arrhythmia	25	10.7
